# Adverse Drug Reaction due to Antidepressants among Patients with Depression in a Private Psychiatric Hospital of Nepal

**DOI:** 10.1155/2020/6682928

**Published:** 2020-11-16

**Authors:** Sabina Sankhi, Nirmal Raj Marasine, Saroj Sankhi, Rajendra Lamichhane

**Affiliations:** ^1^Pharmaceutical Sciences Program, School of Health and Allied Sciences, Pokhara University, Pokhara-30, Kaski, Nepal; ^2^Department of Veterinary Microbiology and Parasitology, Faculty of Animal Science, Veterinary Science and Fisheries, Agriculture and Forest University, Rampur, Chitwan, Nepal; ^3^Department of Public Health, Asian College for Advance Studies, Lalitpur, Nepal

## Abstract

**Background:**

Adverse drug reactions (ADRs) are the known cause of morbidity and mortality, resulting in increased hospitalization, healthcare costs, complications, and decreased adherence in patients with depression. This study is aimed at evaluating adverse drug reactions due to antidepressants among patients with depression in the psychiatric hospital of Nepal.

**Methods:**

A hospital-based prospective observational study was conducted among 47 patients using purposive sampling techniques among patients with depression visiting a private psychiatric hospital. The causality, severity, and preventability assessment of reported ADRs was performed using the Naranjo scale, modified Hartwig Siegel's Severity Assessment Scale, and Schumock and Thornton scale, respectively. The data collected were entered into and analyzed using IBM-SPSS 20.0.

**Results:**

The incidence rate of ADR was found to be 4.54%, with females having a higher incidence rate of 5.56%. A total of 54 ADRs were reported. The majority of them were probable (30, 55.55%), moderate (36, 66.66%) and probably preventable (24, 44.44%). Most of the ADRs were managed either by stopping (19, 35.18%) or substituting (19, 35.18%) the suspected drug and 66.66% of the ADRs were recovered. Selective serotonin reuptake inhibitors (SSRIs) were associated with a higher number of ADRs i.e. 34.

**Conclusion:**

The findings highlight the need for regular intensive monitoring of ADRs in psychiatric outpatients by clinical pharmacists for early detection and reduction of risk caused by ADRs associated with antidepressants.

## 1. Introduction

The World Health Organization (WHO) defines adverse drug reactions (ADRs) as “noxious and unintended response that occurs at doses normally used in humans for prophylaxis, diagnosis or therapy of disease, or for the modification of physiological function” [1]. They warrant prevention, specific treatment, dosage regimen alteration, and even withdrawal of drug in the future. Along with the therapeutic effect, almost every drug has side effects or adverse effects [2]. They are the known cause of morbidity and mortality in both inpatient and outpatient settings [3]. The incidence of ADRs among inpatients was reported to be 6.7%, while that of outpatients was reported to be 5-35% [4, 5].

ADRs are very common in patients receiving psychotropic medications and can even occur at normal doses used in the management of the acute and maintenance phases [6]. Antidepressant drugs are the most effective and widely used forms of treatment for depression but this doesn't mean they are free of adverse drug reactions [7]. Depression is a common psychiatric disorder affecting more than 264 million people around the globe. It is a leading cause of worldwide disability and a major contributor to the overall global burden of disease [8]. ADRs are the significant determinants of adherence associated with antidepressant medications and are reported to be high among patients with depression [9]. ADRs impair the quality of life of people, leading to poor adherence to antidepressant medications, which finally results in longer hospitalization, increased healthcare costs, decreased therapeutic outcome, physical morbidity, stigma issues and, in extreme cases, even death [10]. The incidence of ADRs causing hospitalization was found to vary from 0.2 to 41.3%, and health care costs increased by 5-10% [11, 12].

ADR monitoring in the hospital setting is vital because it helps to understand the nature and type of ADRs and to identify high-risk patients for developing ADRs [13]. In developing countries, ADR monitoring is practiced less. For example, the ADR monitoring rate of developed countries is 5%, while that of India is only 1% [14]. This might be due to lack of ADR reporting due to guilt, lack of awareness, motivation, ignorance, training, and time limitations among healthcare personnel [15]. In Nepal, there is paucity of such pharmacovigilance studies, especially in depressive patients under antidepressant medications. ADR monitoring acts as a supporting framework for the development of interventional strategies that will manage, prevent, and reduce the risk of ADRs in patients using antidepressant medications, thereby reducing the healthcare costs [16]. This study focuses on enhancing and strengthening the pharmacovigilance activities in Nepal and establishing the role of clinical pharmacists in ADR monitoring and reporting. Hence, this study is aimed at evaluating adverse drug reactions due to antidepressants among patients with depression in the psychiatric hospital of Nepal.

## 2. Methods

### 2.1. Study Design and Population

A hospital-based prospective observational study was conducted between August 2019 and July 2020 among 47 patients using the purposive sampling technique at B.G. Hospital. This study was approved by the institutional review committee (IRC) of Pokhara University Research Centre (PURC) (Ref.No.18-076-077). Written informed consent was obtained from all the participants prior to their involvement in the study, and all the information was kept confidential.

All patients aged between 18 and 65 years, diagnosed with depression, taking antidepressant medication for at least one month, and having regular visits to B.G. hospital for follow-up and/or medication refill were eligible to be included in the study. Pregnant or lactating mothers, those with a history of psychotic, bipolar disorder or drug abuse, those with cognitive impairment, and those unable to communicate and understand the Nepali language were excluded.

### 2.2. Data Collection and Analysis

A standard data collection form was developed in accordance with the ADR reporting form of the Department of Drug Administration (DDA), Ministry of Health and Population, Nepal [17]. Information on demographics (age, sex), ADR details (the nature of reaction, date of onset), information on suspected medicine (brand and generic name, manufacturer, batch no., dosage form, daily dose, start date, stop date, and reason for use), and additional relevant information (medical history, test result, known allergies, drug interactions) were recorded. The pattern of ADRs thus obtained was analyzed.

The suspected drugs were identified and their causal associations were analyzed using the Naranjo scale [18]. As per the scale, a score of >9 indicated definite ADRs, 5-8 indicated probable ADRs, 1-4 indicated possible ADRs, and 0 indicated doubtful ADRs. The severity of ADRs was analyzed using the modified Hartwig Siegel's Severity Assessment Scale [19], which classifies ADR into mild (1-2 points), moderate (3-4 points) and severe (5-7 points). The preventability assessment was performed using the Schumock and Thornton scale [20], which classifies the ADRs into definitely preventable, probably preventable and not preventable. The data collected were entered into and analyzed using Statistical Package for the Social Sciences (SPSS) software version 20.0 for Windows (IBM Corporation, Armonk, NY, USA).

## 3. Results

A total of 1035 (550 male and 485 female) depressive patients visited B.G. Hospital, Nepal, during the study period, of which 54 ADRs were detected among 47 patients. The overall ADR incidence rate was 4.54%. The male-to-female ratio was 1 : 1.35. Females experienced a higher incidence of ADRs (5.56%) than males (3.63%). The majority of the patients (17, 36.17%) were in the age group of 31-40 years, as shown in Table 1.

Based on the Naranjo score, approximately 30 (55.55%) of the reported ADRs were probable and 24 (44.44%) were possible in nature. The overall severity assessments showed that the majority of the reported reactions were moderate (36, 66.66%), followed by mild (13, 24.07%) and severe (5, 9.25%) reactions, respectively. Assessment of the preventability of reported ADRs showed that 24 (44.44%) of the reported ADRs were probably preventable, 18 (33.3%) were definitely preventable and 12 (22.22%) were not preventable.

Of the reported ADRs, the suspected drug was stopped in 19 (35.18%) of the ADRs, followed by drug substitution in another 19 (35.18%) of the ADRs. Similarly, in 7 (12.96%) ADRs, another medicine was added to the patients prescription for their effective management, whereas the usual dose of drug was reduced in 9 (16.66%) of the ADRs. Likewise, in the case of outcome of the ADR management, 66.66% of the ADRs were recovered and 33.33% were in the recovering phase, as depicted in Table 2.

The most common antidepressants causing ADRs and their reactions are illustrated in Table 3. Selective serotonin reuptake inhibitors (SSRIs) were associated with more ADRs (34, 62.96%), followed by tricyclic antidepressants (TCAs) (14, 25.92%).

## 4. Discussion

Our study evaluated adverse drug reactions due to antidepressants among depressive patients in the psychiatric hospital of Nepal over one year. The study revealed the overall incidence rate of ADR to be 4.54%, where females showed a higher incidence rate than males, mostly belonging to the age group of 31-40 years. These data are comparable to those reported from India, which showed the overall incidence rate to be 0.69% and that of males and females to be 0.52% and 0.94%, respectively [21]. The higher incidence rate seen in our study might be due to evaluation carried out for comparatively less duration and in fewer patients than that of the Indian study, which was carried out for 2 years in 9701 patients. Additionally, the study included all psychiatric patients and overall drugs, whereas we included only depressive patients using antidepressants. Conversely, other studies have reported an incidence rate of 5.01–21.45% in psychiatric outpatients [6, 22]. The lower rate in our study suggests that many ADRs might have left unreported, which might be due to underreporting or non-interest of psychiatrists/physicians in reporting the established drug interactions in a reporting system [21]. The higher incidence rate of ADRs in females showed consistency with other previous studies [21, 23]. This might be due to pharmacokinetic, hormonal and immunological differences among genders in terms of the use of medications [21]. Lean body mass, reduced hepatic clearance and difference in the metabolic rate due to difference in the activity of cytochrome P450 might be the other reasons [24].

This study showed that most of the reported ADRs were probable and moderate in nature. This was well supported by a previous study [25] and opposed by another study, where the highest number of suspected ADRs was possible and mild in nature [26]. Additionally, a study conducted in Germany depicted severe ADRs due to antidepressants in 1.4% of psychiatric patients under medication [27]. Likewise, the majority of the ADRs were “probably preventable” type, which was consistent with another study, where almost all ADRs were “probably preventable” type except one, which was “not preventable” type [28]. Moreover, a previous study from the United States where 94 ADRs were suspected illustrated that only 19 of them were “preventable” type ADRs [29]. A study from Italy mentioned that a change in original therapy is the best way to improve ADRs and clinical conditions of the patients [30]. In this study, most of the reported ADRs required either stoppage of the suspected drug or substitution by others for their effective management. These changes might be the reason for the recovery of 66.66% of the reported ADRs seen in the study. A similar study from UAE reported the recovery of 46.4% of ADRs through withdrawal of the suspected drug [13].

Among the antidepressants, SSRIs were found to be primarily associated with more ADRs than other drug classes. ADRs such as gastritis, sedation, weight gain, oral ulcer, restlessness, erectile dysfunction, and tremor were more common in patients taking SSRIs followed by TCAs. These findings were well supported by two other studies from the past [13, 21]. This might be explained on the basis of the mechanism of action of SSRIs, where stimulation of 5-HT3 receptors in the central and peripheral nervous systems causes gastrointestinal reactions such as gastritis, oral ulcers, and diarrhea. The excessive activity at spinal 5-HT2 receptors leads to erectile dysfunction and delay in ejaculation, and overstimulation of 5-HT2 receptors in the brain results in insomnia, anxiety, irritation, worsening of symptoms of depression, decreased libido, etc. [7]. In addition, the antagonism of 5HT2c, H1 receptor, hyperprolactinemia, and increase in the serum leptin level consequence in weight gain in patients taking antipsychotic medications [31].

The major limitation of this study was the short study period and the study subjects were confined to the outpatient department of a single centre. To identify a wide spectrum of ADRs of different drug classes, a study duration of more than 1 year could be more effective. Besides this, the study provides insight into the adverse effects of antidepressants in depressive patients of western Nepal. The data obtained could be a baseline for making comparisons with similar studies from different parts of the country. Furthermore, the findings might be beneficial in reducing the morbidity rate, making drug therapy safe, and more rational by formulating policies for the effective management of ADRs in psychiatric hospitals in Nepal.

## 5. Conclusion

This study suggests that the overall incidence of ADRs due to antidepressants was 4.54%, with females having a higher incidence rate. The majority of the reported ADRs were probable, moderate in nature, and probably preventable. A good number of ADRs were recovered upon stoppage or substitution of the suspected drug, and SSRIs were the main drug with a high number of reported ADRs. These findings highlight the need for regular intensive monitoring of ADRs in psychiatric outpatients by clinical pharmacists for early detection and reduction of risk caused by ADRs. This, as a consequence, may improve quality of care, reduce total healthcare costs, and therefore enhance adherence among patients with depression.

## Figures and Tables

**Table 1:** Baseline characteristics of the study population.

Characteristics	Category	n (%)
Sex	Male	20 (42.53)
	Female	27 (57.44)
Age	18 -30	13 (27.65)
	31-40	17 (36.17)
	41-50	11 (23.40)
	51-65	6 (12.76)

**Table 2:** Summary of adverse drug reactions based on severity, preventability, management, and outcome.

Characteristics	Category	n (%)
Causality (Naranjo's algorithm scale)	Definite	0 (0.00)
	Probable	30 (55.55)
	Possible	24 (44.44)
	Doubtful	0 (0.00)
Severity (modified Hartwig and Siegel scale)	Mild (1-2 points),	13 (24.07)
	Moderate (3-4 points)	36 (66.66)
	Severe (5-7 points)	5 (9.25)
Preventability (modified Schumock and Thornton scale)	Definitely preventable	18 (33.33)
	Probably preventable	24 (44.44)
	Not preventable	12 (22.22)
Management	Stopped the medication	19 (35.18)
	Substituted another drug	19 (35.18)
	Reduced the dose	9 (16.66)
	Added another drug	7 (12.96)
Outcome of management	Recovered	36 (66.66)
	Recovering	18 (33.33)
	Fatal	0 (0.00)

**Table 3:** Antidepressant classes commonly associated with adverse drug reactions.

Drug class	WHO-ATC code	No. of ADRs	ADRs
SSRIs	N06AB	34	Gastritis (5), sedation (4), oral ulcer (3), restlessness (3), erectile dysfunction (2), tremor (2), anxiety (2), insomnia (1), dizziness (1), headache (1), weight gain (3), vomiting (1), abdominal discomfort (1), abdominal distension (1), diarrhea (1), postural hypotension (1), sweating (1), problem with urination (1)
TCAs	N06AA	14	Sedation (3), gastritis (2), weight gain (2), dryness of mouth (2), indigestion (2), blurred vision (1), headache (1), restlessness (1)
^∗^Other antidepressants	N06AX	6	Dry mouth (1),weight gain (1), dizziness (1), tremor (1), nausea (1), anxiety (1)

Abbreviation: WHO-ATC: World Health Organization-anatomical therapeutic classification; ADR: adverse drug reaction; SSRIs: selective serotonin reuptake inhibitors; SNRIs: serotonin norepinephrine reuptake inhibitors; TCA: tricyclic antidepressants. ^∗^Other antidepressants: duloxetine, venlafaxine, bupropion, mirtazapine.

## Data Availability

The raw data used to support the findings of this study are made available from the corresponding author upon reasonable request.
